# Patterns of Irregular Burials in Western Europe (1^st^-5^th^ Century A.D.)

**DOI:** 10.1371/journal.pone.0130616

**Published:** 2015-06-26

**Authors:** Marco Milella, Valentina Mariotti, Maria Giovanna Belcastro, Christopher J. Knüsel

**Affiliations:** 1 Anthropological Institute and Museum, University of Zurich, Winterthurerstrasse 190, 8057 Zurich, Switzerland; 2 Laboratorio di Bioarcheologia e Osteologia Forense, Dipartimento di Scienze Biologiche, Geologiche e Ambientali, Alma Mater Studiorum Università di Bologna, Via Selmi 3, 40126, Bologna, Italy; 3 UMR 7268 ADÉS–Anthropologie Bioculturelle, Droit, Ethique et Santé CNRS / Université d’Aix-Marseille / EFS Faculté de Médecine–Secteur Nord, CS80011, Boulevard Pierre Dramard 13344, Marseille Cedex 15, France; 4 Centro Fermi, Piazza del Viminale 1, 00184 Roma, Italy; 5 UMR5199 PACEA, Université de Bordeaux, Bâtiment B8, Allée Geoffroy Saint Hilaire, CS 50023, Pessac Cedex 33615, France; University of Otago, NEW ZEALAND

## Abstract

**Background:**

Irregular burials (IB—burials showing features that contrast with the majority of others in their geographic and chronological context) have been the focus of archaeological study because of their relative rarity and enigmatic appearance. Interpretations of IB often refer to supposed fear of the dead or to social processes taking place in time-specific contexts. However, a comprehensive and quantitative analysis of IB for various geographical contexts is still lacking, a fact that hampers any discussion of these burials on a larger scale.

**Methods:**

Here, we collected a bibliographic dataset of 375 IB from both Britain and Continental Europe, altogether spanning a time period from the 1^st^ to the 5^th^ century AD. Each burial has been coded according to ten dichotomous variables, further analyzed by means of chi-squared tests on absolute frequencies, non-metric multidimensional scaling, and cluster analysis.

**Results:**

Even acknowledging the limits of this study, and in particular the bias represented by the available literature, our results point to interesting patterns. Geographically, IB show a contrast between Britain and Continental Europe, possibly related to historical processes specific to these regions. Different types of IB (especially prone depositions and depositions with the cephalic extremity displaced) present a series of characteristics and associations between features that permit a more detailed conceptualization of these occurrences from a socio-cultural perspective that aids to elucidate their funerary meaning.

**Conclusions and Significance:**

Altogether, the present work stresses the variability of IB, and the need to contextualize them in a proper archaeological and historical context. It contributes to the discussion of IB by providing a specific geographic and chronological frame of reference that supports a series of hypotheses about the cultural processes possibly underlying their occurrence.

## Introduction

Irregular burials (IB—a definition preferred throughout this paper to the more commonly used but less neutral “deviant burials”–[1]), refer to mortuary practices “…different from the normative burial ritual of the respective period, region and/or cemetery” [2]. If focusing only on inhumation burials (and therefore not considering cremation rites, which is beyond the scope of this paper) and Western Europe, from the Roman period onward, customary burials are represented by depositions in an extended, supine position, with no further modification of the body or skeleton. For the same chronological span, and for a wide geographical area, reported cases of IB for the most part relate to prone burials and inhumations with evidence of modification of the corpse or skeleton (beheading and other mutilations, including transfixion of the body with nails) [3–6]. Additionally, irregular burials can be characterized by the unusual orientation of the once corpse in the grave, and by the anomalous orientation and location of the grave itself [2,4]. Note that what actually defines an IB must also be contextualized on the basis of additional features of the burial and the buried individual (e.g. presence of grave goods, presence of grave furniture (e.g. coffins, boxes), age and sex of the individuals).

IB have attracted the interest of a number of scholars concerned with their possible meaning from a funerary and, more general, social perspective, with early discussions of these findings largely influenced by ethnographic studies documenting inter-population similarities in funerary behavior (see 174–179 in [7,8] as well as [9,10]). Particularly influential were hypotheses that linked the fear of the deceased to the preventive function of certain funerary rituals for the most part documented in ethnological societies [11,12]. The subsequent inclusion of both a sociological and anthropological perspective in archaeological and bioarchaeological research [13–16] has markedly influenced the discussion of funerary contexts [17]. The renewed focus on the social dimension of funerary contexts has led to new interpretation of findings that, in various geographical and chronological contexts, are characterized by specific treatments of the corpse or skeleton. Examples of this line of enquiry include works dealing with the link between social constructions of deviancy and specific funerary treatments [18], as well as on the possible relationship between cultural and political perspectives on treatments of the body and headhunting in Iron Age Europe [19] and patterns of identity, social structure and social memory during the Natufian and Pre-Pottery Neolithic of Southwest Asia [20,21] and the Iberomaurusian of North Africa [22–24]. In this context, IB have been investigated with appropriate archaeological methods and renewed attention to and thorough study of the osteological remains [3,4,25–28]. Altogether, these works represent a second, more critical phase of studies of IB. Nonetheless, studies of IB are still hampered by the patchiness of the archaeological evidence and the ambiguity of the historical sources. The inability to study the variability of IB in relatively large samples, together with the almost total absence of information about the possible meaning of the observed treatments in the context of a specific cultural framework (see also [29]), are the greatest obstacles to interpretations of IB. In addition, meta-analyses and comparisons between studies are hampered by the often cursory archaeological documentation, an issue affecting especially the oldest archaeological reports (cf. [30]). Detailed reviews of IB are indeed available only for Romano-British and Anglo-Saxon contexts (see 89–93 in [3,4] as well as[31]). On the other hand, few syntheses are available for Continental Europe (but see [32]).

Romano-British and Anglo-Saxon findings have been linked to necrophobic beliefs (cf.[31]) as well as to magico-ritual behaviors, forms of “overkill”, executions, and, in a broad sense, marginalization processes (see 89–93 in [4] as well as[5]). Other interpretations include the possible link with ritual practices dating to (at least as early as) the European Iron Age [33,34] and coeval with cultural crises following the spread of Christianity in the Roman provinces [33]. According to Philpott [3] decapitated burials would be consistent with the totemic force of the head in so-called Celtic cultures (but see the objections raised by Armit [19] about the concept of a "Celtic" cult of the head]. The “necrophobic hypothesis” linking IB to supposed fearful attitudes toward the dead underlies various studies from Continental Europe. At Avenches (Canton Vaud, Switzerland), protective practices against premature death have been cited as a possible explanation for the correlation between young age at death and prone inhumations [35]. Similar hypotheses have been posited for evidence from Germany, Austria and Denmark [7,8,9,36,37–39] and, more recently, for a larger set of geographical contexts [40–42]. Altogether, old and recent studies on IB are largely affected by the same issues: (a) positing of hypotheses that are not clearly supported by the available historical and/or literary sources, (b) a marked tendency to rely on ethnographic sources (i.e. the “necrophobic hypothesis”), and (c) a neglect of large-scale demic and socio-cultural processes possibly underlying the observed patterns.

In order to address these shortcomings, we synthesized the data available for Western Europe from a bibliographic dataset of the readily available publications of European IB dated to the first to fifth centuries A.D., and then subjected these data to multivariate analysis. We address the following questions: (a) Are the most frequent typologies of IB characterized by a distinct geographical pattern? (b) Are specific IB (e.g. prone, decapitated burials) characterized by distinct features (presence of grave goods, coffins or other grave features)? Being fully conscious of the dictum that every synthesis implies a significant loss of information, we consider a time frame encompassing events such as Romanization, fall of the Roman Empire, “barbarian invasions”, and the spread of Christianity; note also that under the label “grave goods” many different objects with different meanings are included, from those related to funerary libations to apotropaic amulets. The rationale for this approach is to provide a general framework within which specific occurrences could be analyzed and further contextualized as a starting point for future studies.

## Materials and Methods

We collected a bibliographic dataset for a total of 406 IB selected according to the following criteria:

- Type of burial limited to single inhumations
- Dating between the 1^st^ and the 5^th^ centuries AD
- Geographical coverage being restricted to Western Europe
- Archaeological and anthropological evidence consistent with the following characteristics: prone position of the body, evidence of decapitation, other types of mutilation, and/or transfixion of the body by means of nails and close association of nails to the skeleton (e.g. nails in the oral cavity).

**Fig 1:**
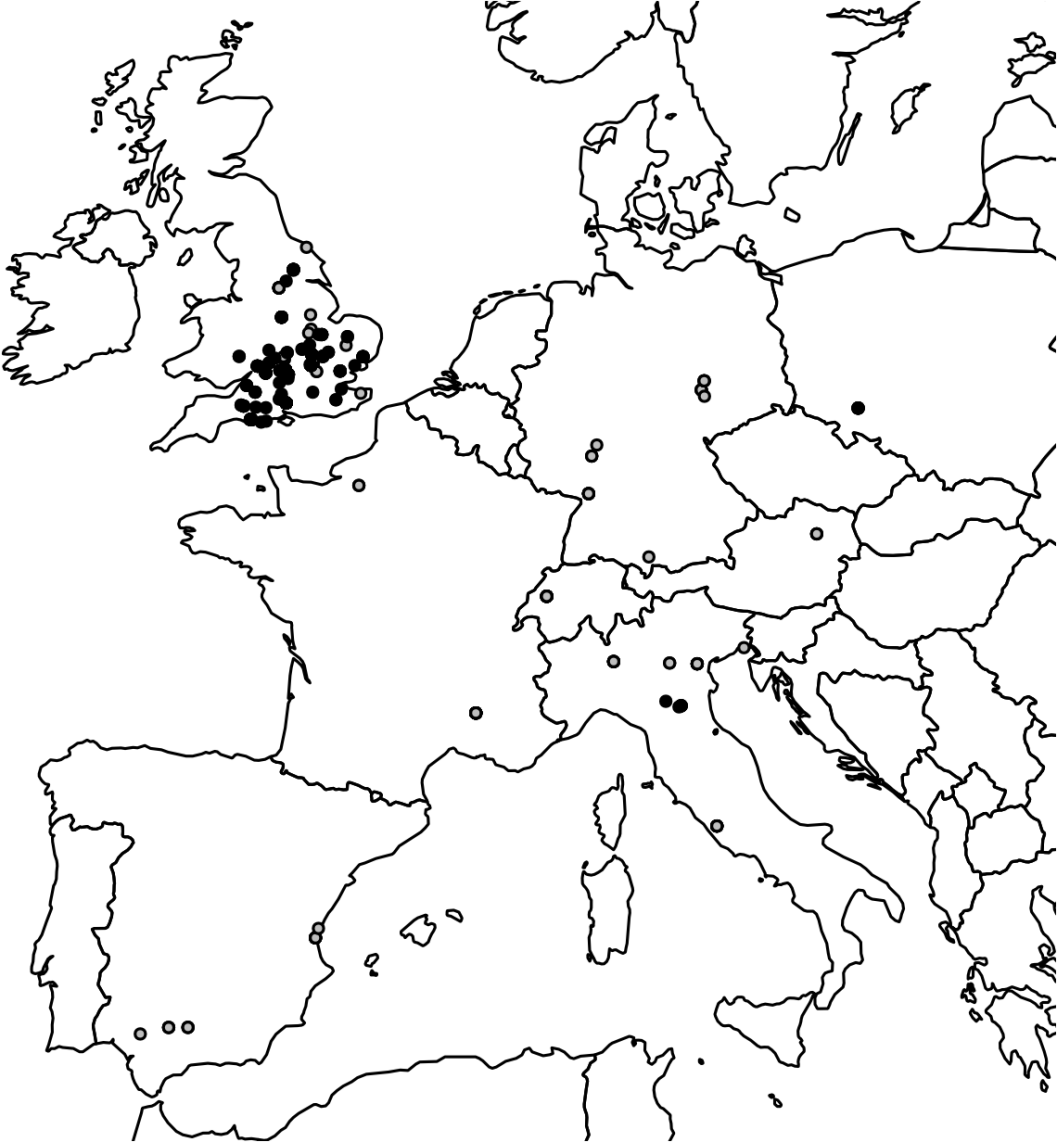
Geographical distribution of the prone burials and burials with the cephalic extremity displaced. Each dot represents a site. Grey and black dots indicate, respectively, prone burials and burials with the cephalic extremity displaced. Note the wide distribution of prone burials in both Continental Europe and Britain, and the cluster of burials with the cephalic extremity displaced in the latter.

Each burial was scored according to ten variables (Table 1). Variables were selected in order to summarize the irregular features of each case as well as to maximize the comparability between cases while minimizing the bias introduced by the lax or unclear terminology employed in older publications. Accordingly, information on sex, age-at-death and pathological conditions was not included due to an overall lack of pertinent data or due to dubious attributions. Only the presence of subadults, which can be confidently assessed even from older publication, was considered. The same rationale led to the exclusion of features often interpreted in the context of IB (e.g. skeletons covered by stones, presence of amulets [4,8] or position of the grave with respect to the burial area [4]). The exclusion of cases for which variables were not reported led to a reduction of the initial sample to 375 inhumations (Fig 1 and S1 Table).

**Table 1:** Definition of the variables used in this study.

Cephalic extremity displaced: post-cranial skeleton in anatomical position but a) cranium or skull (cranium and mandible, and possibly including superior cervical vertebrae) present in the cephalic area, but voluntarily displaced from strictly anatomical position; b) cranium or skull (cranium and mandible, and possibly including superior cervical vertebrae) present, but far from the cephalic area. In both cases a) and b) the individual could have been decapitated, either *intra vitam* (e.g. execution) or *post mortem* (e.g. manipulation of the cadaver). Note that possible cut marks related to decapitation are not always observable due to the preservation status of the skeletal material, or their presence/absence is not reported. In some cases, the possibility of an intentional displacement of the head/skull after a period of interment cannot be ruled out. *
Cephalic extremity missing: absence of the cranium and possibly of the mandible and superior cervical vertebrae but not due to taphonomic causes. The absence of elements of the cephalic extremity can be linked to both decapitation and post-mortem removal.
Post-cranial bones missing: displacement or absence of elements of the post-cranial skeleton due to a) intentional retrieval from the grave, possibly after a period of interment; b) peri-mortem mutilation (presence of cut marks or evident displacement, e.g. foot elements near the cephalic area of the burial). Only explicitly described cases are considered.
Prone: the entire skeleton lies ventrally. Hyperflexed and semi-prone positions are not considered.
Subadult: age at death less than 14 years.
Grave goods: presence of objects deliberately included in the burial not related to clothes.
Nails: presence of nails in the burial space not related to burial structures. Nails could transfix body parts or be closely associated with the skeleton.
Animals: presence of zoological remains deliberately included in the grave.
Footwear: presence of archaeological evidences of footwear in the burial space.
Coffin: presence of sarcophagi/container of any material.

* Due to misuse and inconsistent use of terms in the literature it is not always possible to assess whether both cranium and mandible were involved or solely the cranium, as well as the possible presence of uppermost cervical vertebrae.

Data were then analyzed following two strategies. First, we explored the geographical distribution of IB, by focusing on the most commonly represented types of findings in order to obtain a satisfactory sample size and geographical representation. Second, we investigated the co-occurrence of the ten variables separately in Britain (N = 266) and Continental Europe (N = 109). The reasons for such subdivision are, on one hand, the need to maximize the size of the relatively poor and patchy sample from Continental Europe (see below), which, if considered by sub-regions, would be too small and, on the other hand, the different historical processes attested in these main regions (e.g. Romanization, Christianization). Relative frequencies of IB for each site were not considered due to: (a) the frequent lack of such information, and (b) the statistical bias represented by only partially excavated contexts. The frequent lack of details for each variable meant scoring them dichotomously as present (1) or absent (0). Differences between Britain and Continental Europe were explored by means of a chi-square test on absolute frequencies of burial features. Moreover, in order to explore any possible patterns underlying the variables in our dataset we analyzed the latter, separately for Britain and Continental Europe, by means of non-metric multidimensional scaling (NMDS). In brief, in NMDS, a dataset is projected into a low-dimensional space (two or three dimensions) after being converted to a distance matrix. The Euclidean distances in the plot reflect the distances calculated between each point, after a ranking procedure [43]. In our case, the conversion of the dataset into a distance matrix was computed by using the Jaccard similarity index [44], and the multidimensional space was defined by two dimensions. In addition to NMDS, the data were processed by means of UPGMA (Unweighted Pair Group Method with Arithmetic Mean) cluster analysis on Euclidean distances with bootstrapping (N bootstrap replicates = 1000). Statistical analyses were performed with PAST [45], setting alpha at 0.05.

## Results

**Fig 2:**
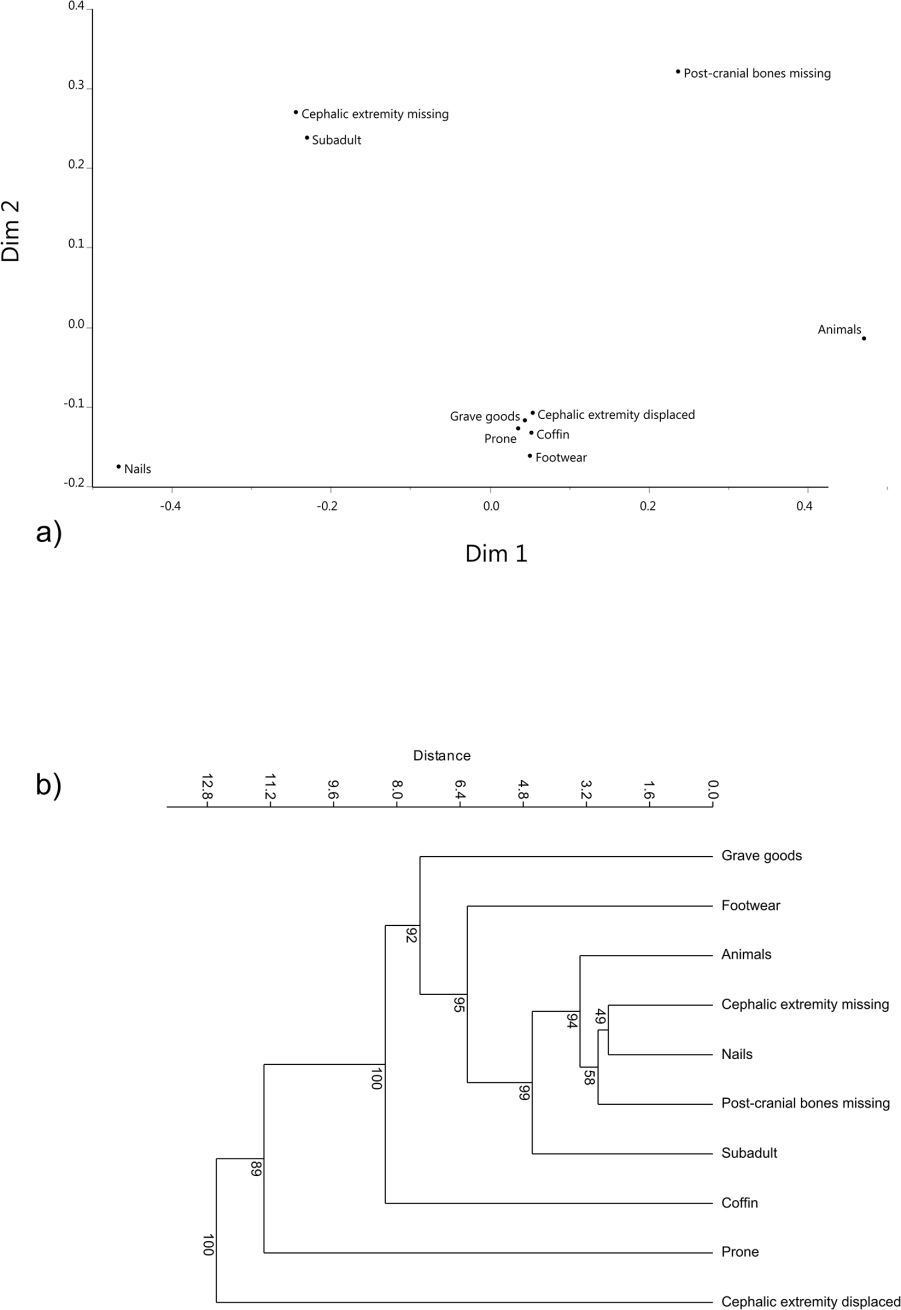
Britain. a) Non-metric multi-dimensional scaling of burial similarities. Note the association of grave furnishing (grave goods and coffins) with prone burials and burials with the cephalic extremity displaced. b) Cluster analysis of burial features.

Of the 375 IB studied in this work, 71% (266) are from Britain and 29% (109) from Continental Europe. Our dataset is strongly biased toward prone burials and burials with the cephalic extremity displaced (Table 2). Accordingly, we decided to focus on these features in order to explore their geographical distribution. Prone burials show a wide distribution that covers the entire geographical area examined, while burials with the cephalic extremity displaced are almost always found in southern Britain, with only few examples from Continental Europe (Fig 1). Other differences between Britain and Continental Europe are illustrated by the chi-squared test on single variables (Table 2). Continental Europe presents a significantly higher frequency of prone burials, presence of grave goods, presence of a coffin, and presence of nails. On the other hand, Britain is characterized by a significantly higher frequency of displacement of the cephalic extremity. As far as the association between variables is concerned, Continental Europe shows significantly higher associations between prone burials and the presence of grave goods and coffins, respectively (Table 2), while Britain shows significantly higher frequencies of prone burials with the cephalic extremity displaced and of the latter with grave goods, coffins, and footwear. NMDS demonstrates specific trends in both Britain and Continental Europe. In Britain (Fig 2A), the most frequent types of features occupy the lower part of the center of the plot, and exhibit a position relative to each other consistent with their pattern of co-occurrence. Accordingly, prone burials and displacement of cephalic extremity are not isolated from each other, a pattern reflecting their partial association in Britain. Presence of grave goods, presence of coffins, and footwear are associated with both cephalic extremity displacement and prone burials in Britain.

**Table 2:** Frequency of each variable and combined variables in Britain and Continental Europe.

	Britain			Continent			
	total	presence	presence%	total	presence	presence%	p
Prone	266	127	47.7	109	100	91.7	**<0.001**
CD	266	156	59	109	3	2.8	**0.00**
CM	266	5	1.9	109	2	1.8	0.98
Grave goods	248	44	17.7	93	29	31.2	**0.01**
Footwear	247	30	12.1	92	10	10.9	0.75
Animals	266	8	3	109	3	2.8	0.89
Coffin	239	57	23.8	97	34	35.1	**0.04**
Subadult	266	18	6.8	108	8	7.4	0.83
Nails	266	2	0.75	109	6	5.5	**0.00**
PM	266	5	1.9	109	6	5.5	0.06
Prone and grave goods	248	17	6.9	93	27	29	**<0.001**
Prone and CD	266	17	6.4	109	1	0.9	**0.02**
Prone and coffin	239	19	7.9	97	32	33	**<0.001**
Prone and footwear	247	15	6.1	92	9	9.8	0.24
Prone and subadult	266	9	3.4	108	7	6.5	0.18
Prone and PM	266	2	0.8	109	1	0.9	0.87
Prone and CM	266	0	0	109	1	0.9	0.12
Prone and animals	266	3	1.1	109	3	2.8	0.26
Prone and Nails	266	2	0.8	109	3	2.8	0.13
CD and grave goods	248	30	12.1	93	0	0	**<0.001**
CD and coffin	239	41	17.2	97	0	0	**<0.001**
CD and footwear	247	17	6.9	92	1	1.1	**0.03**
CD and subadults	266	9	3.4	108	0	0	0.05
CD and nails	266	0	0	109	1	0.9	0.12
CD and animals	266	2	0.8	109	0	0	0.37
CD and PM	266	4	1.5	109	2	1.8	0.82

Significant differences between the two areas are reported in bold. CD = Cephalic extremity displaced; CM = Cephalic extremity missing; PM = Postcranial bones missing.

**Fig 3:**
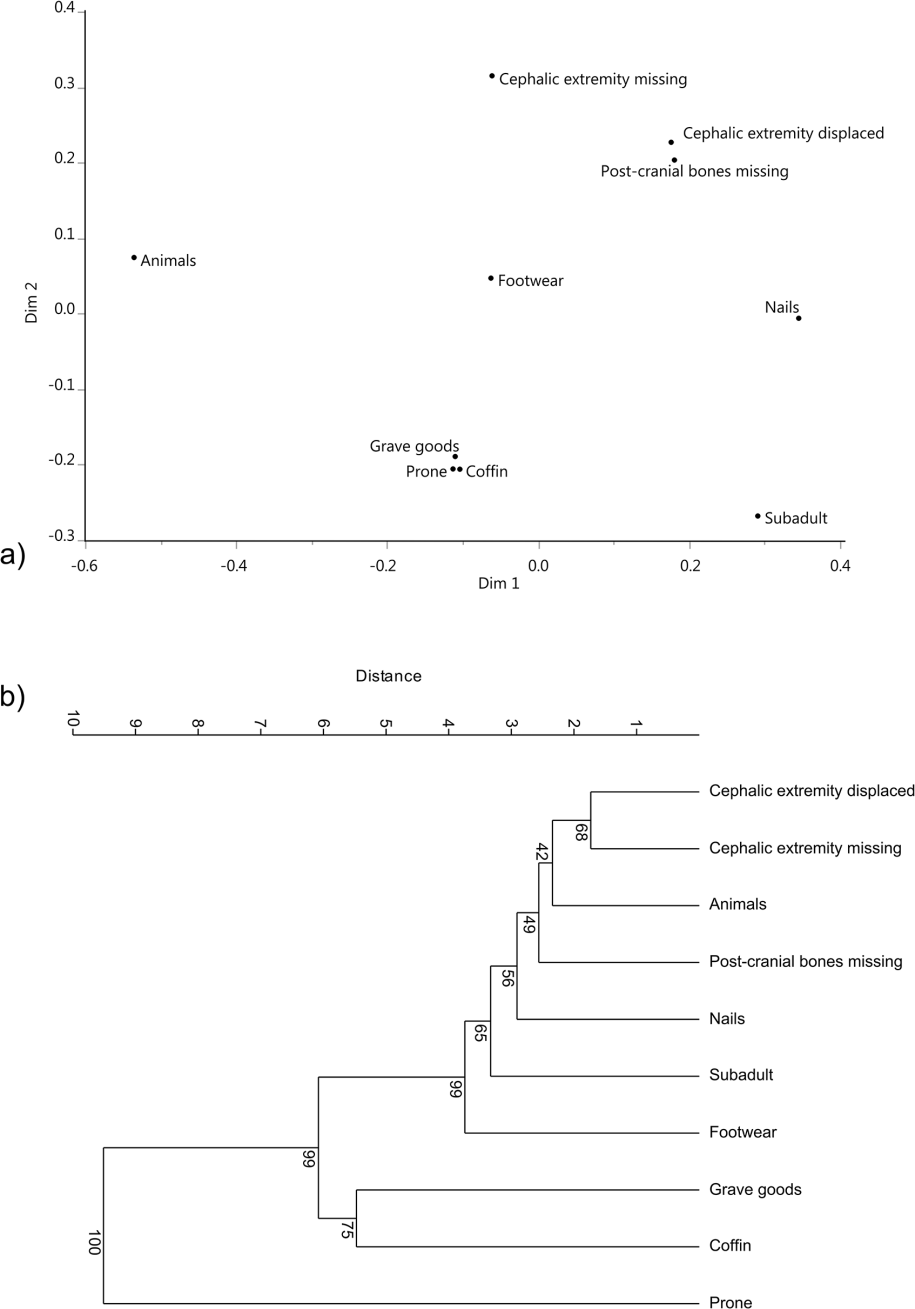
Continental Europe. a) Non-metric multi-dimensional scaling of burial similarities. Note the separation (lack of co-occurrence) between cephalic extremity displacement and prone burials, and the association between the latter and grave goods and grave furniture (coffins). b) Cluster analysis of burial features.

Continental Europe (Fig 3A) is characterized by a specific pattern. Displacement of the cephalic extremity is among the features occupying a peripheral region of the plot, consistent with their rareness in this dataset. The most frequent variables include: prone position of the body, presence of a coffin, and presence of grave goods (all closely associated).

In both Continental Europe and Britain prone burials and burials with the cephalic extremity displaced are separated for the most part from other classical “deviant” features (i.e. nails, cephalic extremity missing, post-cranial bones missing). The patterns reflected by the NMDS are further illustrated by the cluster analyses computed on single variables. In Britain, the clearest associations are between post-cranial bones missing, presence of nails, cephalic extremity missing, and presence of animal remains (Fig 2B). In Continental Europe, on the other hand, displacement of the cephalic extremity and prone burials are clearly separated, and the former is close to cephalic extremity missing. Presence of grave goods and presence of a coffin are relatively closely associated with each other and are altogether closer to prone burials than to cephalic extremity displaced (Fig 3B).

## Discussion

Before discussing our results, we must highlight some issues that influence such a study, namely:

(i) the characteristics of our dataset, i.e. the quality and quantity of the available documentation; (ii) the choice of the variables to be analyzed; (iii) the time span considered in the study.

- The uneven distribution of IB in the area considered (71% in Britain and 29% in the much more extensive area of Continental Europe) could reflect the intensity of the fieldwork and published documentation rather than a real higher frequency of IB in Britain. However, other possible factors should be taken into account. Many cases of IB can be typically found in excavation reports housed in the archives of cultural heritage institutions, which makes this type of information not widely available. A full review of all cases of IB would indeed require years of research in the institutions preserving the excavation records produced in each European country. In addition, many cases of IB are reported in local journals often using the national languages, and thus of difficult access to the international scientific community. This problem is certainly of a lesser importance for Great Britain, as English is the current scientific language and, as a result, journals that in other countries would be considered as local have a greater chance to reach an international audience.
  We also note the lack of standardization in documenting burial features, a factor that, especially in the case of (but not only) the oldest reports, hampers a comparison between burial patterns. In addition, only a minority of archaeological publications on IB is associated with an anthropological analysis of the human remains, leading to an often significant loss of data of considerable interest for interpretation.
- The choice of the variables is strongly influenced by the possibility to obtain a useful dataset for statistical analyses. The incompleteness of the documentation forced us to exclude some important features (e.g. sex of the individual, burial orientation, relative frequency of IB in each context.) or to ignore important descriptive details (e.g. grave goods considered only as present or absent, rather than by number and kind).
- To obtain a suitable sample size we had to consider an extended time frame, which altogether encompasses five centuries and is characterized by a mosaic of large-scale events (e.g. Romanization, fall of the Roman Empire, barbarian invasions, spread of Christianity), each one arguably playing an important socio-cultural role in the Western Europe, and thus also influencing burial customs.

Keeping in mind these relevant issues, we can propose some reflections about our results in relation to the research questions stated in the Introduction.

Our first research question queried the presence of geographical patterns among the main classes of IB. Results pinpoint a spatial distinction between prone burials and burials with the cephalic extremity displaced, with the former showing the widest distribution (Fig 1). Even acknowledging the limits of our dataset (see above), discarding these differences as the mere by-product of sampling biases is probably unwise, especially in the cases where the higher frequencies are found in Continental Europe, where fewer IB have been reported. The higher frequency of displacement of the cephalic extremity in Britain is also striking, and possibly not only related to the better British documentation, even though the choice of the variables to be analyzed in this work could play a role in this regard. In fact, in the public landfills of Mutina (now Modena, Northern Italy, 3^rd^ c. BC-1^st^ c. AD), a deposit of 80 crania and 6 mandibles was recently excavated, representing quite an enigmatic discovery [46]. Seven crania and 4 mandibles were recovered from a fish-farming basin of the same period, excavated nearby [47]. These have not been considered here as we collected data only on single inhumations, but any inference about different ideologies related to the human head for the considered regions should also take into account such findings.

It is worth posing the question as to whether or not additional processes co-occur in producing the observed patterns. Specifically, one must evaluate the possible consistency of our results with (at least) two hypothetical relationships: (i) a common ethnic and/or cultural background versus local social processes that occurred in culturally and ethnically heterogeneous populations, leading to recurrent patterns in the archaeological evidence within the geographical regions considered; (ii) the presence of complex social networks leading to similarities in the archaeological evidence, despite a heterogeneous cultural background, especially in Continental Europe.

- A direct, unequivocal link between IB and a homogenous ethno-cultural background in the regions considered can be at least in part dismissed on the basis of modern archaeological interpretations [19,48,49]. The odds of a cultural or ethnic interpretation of specific archaeological evidence has recently been advanced with regard to the supposed “Celtic cult of the head”, a phenomenon more likely attributable to local socio-cultural developments, rather than to specific narratives shared throughout Europe and Britain during the Iron Age [19]. In sum, if on one hand a decisive role played by common cultural patterns can be dismissed in our case, it is necessary to stress the complex socio-cultural scenarios taking place in Western Europe for the time period considered. Accordingly, it is still possible to postulate an influence of specific cultural practices that characterize specific contexts on a local basis (see below).
- The presence of a link between the observed archaeological patterns and social networks developing during and after the Iron Age can be at least partially retained, given the complex commercial and demic patterns characterizing Europe between the 7^th^-1^st^ century BC and variously documented archaeologically [50,51]. Large-scale cultural contacts marked by a gradual adoption of material and symbolic contents of external origin by local communities can lead to the adoption of exotic funerary customs and social practices (independently from the specific reasons underlying such adoption) (cf. [52]). We can therefore postulate a link between the wide distribution of practices like prone burials and similar processes. Note, however, that prone burials are found as early as the Upper Paleolithic with a wide geographic distribution [53]. Even acknowledging a role of cultural contacts for the spread of specific funerary behaviors, questions pertaining to the causes leading to the performance of such funerary practices are left unanswered. For the geographical and chronological contexts included in this study these would admittedly encompass a large suite of cultural, social and political variables, arguably specific for each region included in the study; further resolution of these regional associations exceeds the scope of the present analysis. One possible factor seems worth discussing however- the effect of the inclusion of Central Europe and Britain in the Roman Empire between the last centuries BC and the first centuries AD. The spread of the Roman Empire followed a gradual pattern, with its maximum expansion during the beginning of the second century AD. The inclusion of Iron Age communities in the Roman socio-political sphere, with changes affecting politics, religion, and settlement size (i.e. urbanization), was a process burdened with profound social stressors leading to a stage of socio-cultural crisis. Whether funerary treatments such as prone depositions and decapitation were in some way connected to this cultural crisis following such changes is difficult to test in the absence of more precise dating and associated regional archaeological contextual information. Note, however, that similar hypotheses have been proposed by Watts [33] concerning a possible link between the process of Christianization in southern Britain and the practice of prone burial, and, by Armit [19] about the effect of Romanization on the exhibition of human heads in southern France. An effect of Romanization on IB is moreover suggested by its distinct chronological and qualitative developments in Continental Europe and Britain. Britain was only partially included in the Roman Empire around the first half of the first century AD, and one century after the conquest of Gaul [54], and the Romanization process was for the most part limited to main urban centers [55]. Accordingly, the contrast observed between these two areas with regard to the type and number of IB could be at least partially related, beyond differences in archaeological coverage, to distinct processes and effects characterizing the Romanization of these regions. Unfortunately, the reported dating of the archaeological sites considered in this work refers to large time spans (cf. S1 Table), preventing the assessment of possible temporal trends.

It is also interesting to acknowledge the interpretation proposed by Faull [56] with regard to Anglo-Saxon “crouched” (i.e. probably flexed on the side) burials as a form of ethnic distinction adopted by local Britons. Though the inclusion of ethnic variables in the discussions of funerary customs is nowadays seen as at least problematic [19,57], it is nonetheless possible that, in some specific contexts, IB were correlated with patterns of social and cultural affiliation (see for example [33]).

While prone burials and displacement of the cephalic extremity are the focus of this study due to their relatively large sample size and wide geographical distribution, other types of findings are interesting because of their rarity and specific spatial distribution. The best example comes with those burials characterized by traces of transfixion of the corpse or skeleton by nails or by the close association of these objects to the skeleton. In our dataset this type of evidence is represented by a total of eight cases, half of them from Italy. The possible link between this evidence and forms of crucifixion is contradicted by the specific location of the nails, which overall is not consistent with their use in these or other forms of execution (for a review see [6,58,59]). On the other hand, metal nails are known to have been objects characterized in the Roman world by specific magico-ritual meanings, as demonstrated archaeologically by their inclusion in burials as a possible form of amulet [60,61]. As skeletons transfixed with nails *intra vitam*- interpreted in the context of torture- have been reported in Greece from the 2^nd^-1^st^ c. BC [59], we can suggest a Mediterranean origin for this kind of evidence. Note, however, that no clear interpretation can be formulated about the possible meaning of the practice of fixing nails on bodies, given the lack of sources describing this specific practice, and the patchy available archaeological evidence (see also [6]). Accordingly, other possible interpretations can be considered besides the one linking this evidence to the “fear of the dead”.

Our second question pertains to the characteristics of different types of IB. The comparison of the associations of the various variables considered with displacement of the cephalic extremity and prone burials, respectively, between Britain and Continental Europe provides results that are related to the high frequency of prone burials and the few cases of displacement of the cephalic extremity in Continental Europe. Instead, it is interesting to note that, in Britain, grave goods and coffins are more closely associated with cephalic extremity displacement than with prone burials. Differing meanings of “decapitation” and prone burial are thus consistent with our data. Indeed, assuming a link between the presence of grave goods and coffins and a sort of respect for the dead, our results seem to support the hypothesis of a denigratory meaning for prone deposition rather than for burials with the cephalic extremity displaced. Reynolds [4] accepts a denigratory meaning for decapitation. It is possible, however, that the meaning of this practice was rather complex and variable, representing not only aggressive or denigratory acts, but also, in specific instances, more positive or respectful attitudes and beliefs (see for example [19,62,63]) and perhaps a sequence of events that included both emotive responses (see [64]). Besides, note that the choice to score grave furnishings dichotomously is likely to mask quantitative and qualitative patterns possibly correlated with largely different motivations. It is also possible that the type of grave furnishings was independent from the presence or absence of an “irregular” treatment of the deceased but was, rather, consistent with the general trends observed for a specific chronological span and geographical context (cf. [3]). Finally, note that an interpretation of decapitation is further complicated by the few cases of missing elements of the cephalic extremity. These, at least in some instances, could be indeed linked to decapitations. The poor representation of this evidence, and its unclear association with other variables, hampers a fuller discussion of its socio-cultural meaning.

Our results show that cephalic extremity displacement and prone position are rarely associated in Britain, where these two practices are both present in large numbers (Table 2). Also Boylston et al. [34] stressed the apparent independence of decapitation and prone deposition at the Romano-British site of Kempston (UK), positing as an explanation different ritual or social meanings for the two procedures. Note that Reynolds [4] points to a lack of co-occurrence between these procedures for the Anglo-Saxon period.

Our results also suggest an association between missing post-cranial bones and cephalic extremity displacement (possibly due to decapitation, cf. Table 1), especially in Britain, where four out five burials with post-cranial bones missing also show evidence for cranial extremity displacement as well (two out of six in Continental Europe).

## Conclusion

In the present study, we discussed the cultural interpretation of irregular burials on the basis of the geographical distribution and association of features from a large dataset of Western European inhumations dating to the 1^st^ to 5^th^ centuries AD. Our results show a different geographical distribution of displacement of the cephalic extremity and prone deposition, with the former more frequent in Britain and the latter in Continental Europe. They also suggest the presence of specific funerary features characterizing different groups of IB. The overall heterogeneity of IB suggests the unsuitability of a single straightforward interpretation for these occurrences. Nonetheless, we stress that an accurate discussion of IB will be possible only on the basis of more extensive samples and more detailed archaeological and anthropological information (cf.[65]). Note also that in the present work IB were analyzed and discussed on the basis of a specific range of variables, chosen on the basis of the available documentation. Accordingly, our discussion is necessarily limited to those types of evidence and does not pretend to cover all possible types of IB. We also stress the need for standardization in collecting and describing evidence for burial modes. As an example, the term “cephalic extremity” is used here due to the ambiguous terminology of the sources; in the future references to specific skeletal elements (e.g. cranium, mandible, specific cervical vertebrae) should be used to avoid ambiguity that tends to conflate different forms of manipulation of the cephalic extremity and thus different practices (cf. [30]). The publication in largely accessible journals of detailed reviews of IB by geographical area would be an important contribution that would permit rectification of the limitations of this work in future studies. The lack of estimates of relative frequencies of IB should be stressed as a major limitation hampering the analysis of intra- and inter-regional burial variation. Moreover, much further work is required to socio-culturally contextualize IB. An ideal research agenda would place these occurrences in a coherent historical and social framework. Specifically, further research is needed in order to explore the role played by social stratification and urban versus rural burial practices on patterns of IB, and the possible correlation of the latter with social factors such as agency, gender, geographic origin, and life course events.

These considerations, together with the results from our study, further stress the need to discuss IB not as isolated case studies but rather in their multi-dimensional context, not only from an archaeological point of view, but also in a socio-cultural perspective.

## Supporting Information

S1 References Detailed bibliographic for S1 Table. (DOC)

S1 Table Dataset of irregular burials considered in this study. Burial ID: "x" = no burial ID provided; dating: "x >" = dating to at least as early as the x^th^ century.
(XLS)
