# Supplementary material for: Patterns of Irregular Burials in Western Europe (1st-5th Century A.D.)
Source: PLoS One. 2015 Jun 26;10(6):e0130616. doi: 10.1371/journal.pone.0130616 (PMC4482629; doi:10.1371/journal.pone.0130616)
Supplement: S1 References — (DOC) [file pone.0130616.s001.doc]

**S1 References**

Bel V. 2002. Pratiques funéraires du Haut-Empire dans le Midi de la Gaule: la nécropole gallo-romaine du Valladas à Saint-Paul-Trois-Châteaux, Drôme. Lattes: Association pour le Développement de l'Archéologie en Languedoc-Roussillon, Centre de Documentation Archéologique Régional.

Bérard G. 1963. La nécropole gallo-romaine de la Calade, à Cabasse (Var). Deuxième campagne de fouilles (1962). Gallia 21(2):295-306.

Bissoli L. 2001. La popolazione della necropoli: un approccio atropo-archeologico. In: Sannazaro M, editor. La necropoli tardoantica: ricerche archeologiche nei cortili dell'Università cattolica. Milano: Vita e pensiero.

Booth P, Simmonds A, Boyle A, Clough S, Cool H, and Poore D. 2010. The late Roman cemetery at Lankhills, Winchester: Excavations 2000-2005.

Boylston A, Knüsel C, Roberts C, and Dawson M. 2000. Investigation of a Romano-British rural ritual in Bedford, England. Journal of Archaeological Science 27(3):241-254.

Burnham BC, Keppie LJF, Cleary ASE, Hassall MWC, and Tomlin RSO. 2000. Roman Britain in 1999. Britannia 31:371-449.

Carrasco Gómez I, and Doreste Franco D. 2005. Excavaciones arqueológicas en el entorno de la Trinidad: continuidad de un espacio funerario en Sevilla. Romula(4):213-244.

Casal MT, León A, Murillo JF, Sánchez S, García B, Vargas S, Sánchez I, and Pizarro G. 2005. Informe-memoria de la intervención arcqueológica de la urgencia en el sd ss-1 (Parque de Miraflores y Centro de Congresos de Córdoba ). Primera fase. Available at http://hdl.handle.net/10396/2961 (accessed 20th November 2014).

Castella D. 1999. La nécropole gallo-romaine d'Avenches ‘En Chaplix’: fouilles 1987–1992. Volume 1, Étude des sépultures. Lausanne: Cahiers d'archéologie romande.

Cavallini L. 2010. Le sepolture anomale in Italia:dalla lettura tafonomica all’interpretazione del gesto funerario. Pisa: Universitá di Pisa.

Cerdá MP, and García-Prósper E. 2005. Estudio bioantropológico de los restos óseos hallados en el interior de la cloaca de la vía romana del “solar de la morería” de Sagunto. ARSE 39:209-228.

Chambers R. 1976. A Romano-British settlement at Curbridge. Oxoniensia 41(1):38-55.

de Boüard M. 1966. Haute et Basse Normandie. Gallia 24(2):257-273.

Dinwiddy K. 2009. A Late Roman Cemetery at Little Keep, Dorchester, Dorset. Salisbury: Wessex Archaeology.

Dix B, and Taylor S. 1988. Excavations at Bannaventa (Whilton Lodge, Northants) 1970-71. Britannia 19:299-330.

Durost R, Février S, Bohec YL, Lenda S, and Turé I. 2007. Découvertes funéraires gallo-romaines dans le faubourg Saint-Gilles de Langres (Haute-Marne). Revue archéologique de l'Est(Tome 56):349-361.

GarcÍa-Prósper E, and Polo M. 2003. Enterramientos en decúbito prono y un posible preso entre los primeros pobladores de Valencia (siglos II aC-III dC). Dónde estamos:298-316.

Grew FO, Hassall MWC, and Tomlin RSO. 1981. Roman Britain in 1980. Britannia 12:314-396.

Harman M, Molleson TI, and Price J. 1981. Burials, bodies and beheadings in Romano-British and Anglo-Saxon cemeteries. Bulletin of the British Museum of Natural History (Geol) 35:145-188.

Hölbling E. 2008. Das römische Gräberfeld von Pottenbrunn. Untersuchungen zur Bevölkerung des ländlichen Raumes um Aelium Cetium. Die Grabungen des Bundesdenkmalamtes der Jahre 2000-2002. Vienna: Uniwien.

Hunter-Mann K. 2005. 6 Driffield Terrace York. An assessment report on an archaeological excavation. York Archaeological Trust.

Hunter-Mann K. 2006. Romans Lose Their Heads: An Unusual Cemetery at The Mount. http://www.iadb.co.uk/driffield6/index.php (Access date 20/11/2012).

Keppie LJF, Cleary ASE, Hassall MWC, Tomlin RSO, Burnham BC, and Wall HS. 1999. Roman Britain in 1998. Britannia 30:319-386.

Knight WFJ. 1938. A Romano-British site at Bloxham, Oxon. Oxoniensia 2:41-56.

López Flores I. 2007. Resultados antropológicos de campo de la necrópolis romana hallada en c/Bellidos, 18 (Écija, Sevilla). Cæsaraugusta(78):609-630.

Mariotti V, Milella M, Belcastro MG. 2010. Le tombe 6, e 16 della necropoli tardo-antica (V-VI sec. d.C.) di Casalecchio di Reno (BO).Analisi antropologiche. In: Belcastro MG, and Ortalli J, editors. Sepolture anomale Indagini archeologiche e antropologiche dall'epoca classica al Medioevo in Emilia Romagna Giornata di Studi (Castelfranco Emilia, 19 dicembre 2009). Borgo S. Lorenzo (Fi): All'Insegna del Giglio. p 113-119.

Milella M, Mariotti V, and Belcastro MG. 2010. Le tombe 76,109, 161 e 244 della necropoli romano-imperiale (I-III sec. d.C.) della nuova stazione dell'Alta Velocità di Bologna. Analisi antropologiche. In: Belcastro MG, and Ortalli J, editors. Sepolture anomale Indagini archeologiche e antropologiche dall'epoca classica al Medioevo in Emilia Romagna Giornata di Studi (Castelfranco Emilia, 19 dicembre 2009). Borgo S. Lorenzo (Fi): All'Insegna del Giglio. p 94-98.

Milella M, Belcastro MG, and Mariotti V. 2011. Pratiche e rituali funerari devianti nell'Italia romana e tardoantica. In: Labate D, and Locatelli D, editors. L'insediamento etrusco e romano di baggiovara (MO) Le indagini archeologiche e archeometriche. Borgo San Lorenzo (Fi): All'Insegna del Giglio. p 99-102.

Philpott RA. 1991. Burial practices in Roman Britain: a survey of grave treatment and furnishing AD 43-410. Oxford: Tempus Reparatum.

Rankov N, Hassall M, and Tomlin R. 1982. Roman Britain in 1981. Britannia 13(1982):328-395.

Rossi C. 2011. Sepolture in decupito prono nella Patavium di etá imperiale. Pagani e Cristiani, forme ed attestazioni di religiosità del mondo antico in Emilia 10:159-185.

Rossi C, Marini I, Fraternale L, and Canci A. 2012. Face-down burials in the roman world:physical, social and ideological reasons of a deviant practice. Archaeological and anthropological analysis of some examples from x regio (North-Eastern Italy). Poster presented at the 19th Euopean meeting of the Paleopathology Association Lille, August 27 - 29, 2012.

Schleifring J. 1999. Menschliche Skelette in Bauchlage vom kaiserzeitlichen Gräberfeld Groß-Gerau „Auf Esch“. In: Smolla G, Herrmann F-R, Schmidt I, and Verse F, editors. Festschrift für Günter Smolla. Wiesbaden. p 625-635.

Simmonds A, Márquez-Grant N, and Loe L. 2008. Life and death in a Roman city: excavation of a Roman cemetery with a mass grave at 120–122 London Road, Gloucester. Oxford: Oxford Archaeological Unit Ltd.

Taylor M. 1953. Roman Britain in 1952: I. Sites explored. Journal of Roman Studies 43:104-128.

Wahl J, Kokabi M, and Alföldy-Thomas S. 1988. Das römische Gräberfeld von Stettfeld: osteologische Untersuchung der Knochenreste aus dem Gräberfeld. Stuttgart: Kommissionsverlag K. Theiss.

Zotz LF, and Glaser R. 1935. Die spätgermanische Kultur Schlesiens im Gräberfeld von Groß-Sürding: Mit einem menschenkundlichen Beitrag. Leipzig: Kabitzsch.
